# New rhinovirus uncoating intermediate reveals how sodium versus potassium ions influence RNA release

**DOI:** 10.1038/s41598-025-20627-0

**Published:** 2025-10-21

**Authors:** Antonio Real-Hohn, Dieter Blaas

**Affiliations:** Centre for Medical Biochemistry, Max Perutz Labs, Vienna BioCenter, Medical University of Vienna, Vienna, Austria

**Keywords:** Cryoelectron microscopy, RNA, Virus structures

## Abstract

Electron microscopy (EM) of rhinovirus A2 (RV-A2) incubated in Na^+^ phosphate buffer (pH 7.6) for 12 h at 25 °C revealed partial fragmentation, whereas upon incubation in K^+^ phosphate buffer, RV-A2 appeared intact. In buffers adjusted to pH 5.8, these differences became more pronounced; acidic Na^+^ phosphate buffer promoted disintegration of the particles, whereas in acidic K^+^ phosphate buffer, the virus appeared like native. Incubation in the acidic buffers for one hour at 4 °C followed by neutralisation resulted in the respective formation of non-infectious A particles (in Na^+^) and a non-infectious novel uncoating intermediate (in K^+^), which we termed ‘E0 particle’. Negative staining EM revealed phosphotungstate penetration into A particles, but not into E0 particles. Cryo-EM image reconstruction of the E0 particle showed clear differences between A and E0 particles; like native virus, E0 contained VP4 and a pocket factor. Native RV-A2 RNA cores, obtained by gentle proteinase-K digestion in K^+^ and Na^+^ phosphate buffer, respectively, differed in accessibility of dsRNA regions, detected by PaSTRy. Variance in RNA compactness observed in K^+^ versus Na^+^ phosphate buffer was confirmed by rotary shadowing EM; in K^+^ phosphate buffer, the RNA remained condensed while, in Na^+^ phosphate buffer, distinct unfolding stages were apparent.

**Supplementary Information:**

The online version contains supplementary material available at 10.1038/s41598-025-20627-0.

## Introduction

The 169 rhinovirus (RV) types identified to date are a main cause of the common cold. They constitute three species within the genus *Enterovirus* that is part of the large family *picornaviridae* (https://www.picornaviridae.com/ensavirinae/enterovirus/enterovirus.htm). They are icosahedral, about 30 nm in diameter and have a ss(+)RNA genome enclosed within a protein shell composed of 60 copies each of the capsid proteins VP1, VP2, VP3, and VP4; the latter is myristoylated. In many RV types, a ‘pocket factor’, in most cases a myristate, was identified in a hydrophobic pocket lined by amino acid residues mainly contributed by VP1. During infection, the native virus expands and releases the pocket factor and VP4 giving rise to the ‘A particle’. The A particle finally discharges the genomic RNA resulting in the empty B particle. The process can be mimicked to a certain degree in vitro by incubation in buffers of low pH and/or heating to about 50 °C in low ionic-strength buffers^1^. How the RNA leaves the viral protein shell and arrives in the cytosol for infection is still a matter of debate^2–8^.

In previous work Real-Hohn and colleagues discovered that quadruplex-forming G-rich sequences (QGRS) present in the RV-A2 genome within the intact capsid were accessible for the QGRS-binding compound pyridostatin; their accessibility strongly depended on the cations in the incubation buffer^9^. The observed variance in permeability of the virus capsid and/or the quasi-crystalline RNA for pyridostatin suggested subtle conformational differences of the capsid and/or of the genomic RNA in buffers of different ionic composition as had been used in the various in vitro assays described in the literature. This might apply particularly for those picornaviruses that become converted in vitro into stable uncoating intermediates at low pH (e.g. coxsackievirus B3^10^; echovirus 18^6^; enterovirus D68^11^; enterovirus 71^12^; and RV-A2^13^). Of note, the uncoating intermediates reported for the above viruses were generated in different ionic environments at pH ~ 5.8 at zero or low concentration of potassium ions. However, a possible impact of Na^+^ versus K^+^ on structural changes of the virion was not studied in any detail. To our knowledge, an attenuating effect of potassium ions on picornavirus uncoating in vivo was systematically assessed only by Irurzun and Carrasco^14^; they showed that poliovirus uncoating proceeds with lower efficiency in the presence of potassium ions as compared to sodium ions.

Taking advantage of the slow kinetics of conversion of native *Enteroviruses* into A particles allowed for the detection of an intermediate transitory uncoating state described as ‘preparatory state’ or ‘expanded 1 (E1)’ for enterovirus D68 by the Rossmann group^11^. A similar uncoating intermediate was observed in poliovirus by P. N. M. Shah and colleagues^15^. Since the poliovirus shell is stable at low pH, here, the transitory state was obtained by brief heating to 50 °C in neutral low salt buffer for 3 min. By the same token Lee et al.^16^ incubated coxsackievirus B3 at 37 °C for 30 min in acidic buffer containing 100 mM NaCl again resulting in a transitory state pre-A subviral particle. All the above corroborates the idea of picornavirus uncoating occurring via subtle consecutive structural changes of the virion such as progressive expansion of the shell and the genomic RNA core^17^ promoted by changes in the ionic environment^18^. This was demonstrated in RV-A2 by labelling the viral genome within the viral capsid with the fluorescent probe SYTO-82 during temperature ramping^19^; the signal increased when the capsid became permeable for the compound and finally decayed upon release of the genome^8,19^. There are at least four enterovirus conformations; (i) the infectious native virion containing a pocket factor, RNA, and VP1-4, (ii) an expanded (E1) subviral particle very similar to the native virion but with increased permeability, (iii) the A particle containing RNA, and VP1-3, and, finally, (iv) the empty B particle lacking VP4, the pocket factor, and the RNA. It is possible that an additional intermediate missing only the pocket factor exists; in vitro, such a particle was obtained by extraction of native RV-A89 with DMSO; it shows subtle changes in the disposition of the amino acid residues lining the empty hydrophobic pocket^17^ similar to native RV-B14 that lacks a pocket factor^20,21^.

The above conformations of virions seen in vitro under various ionic conditions suggest that similar particles might occur in vivo during infection. However, how to conciliate the in vitro with the in vivo findings where the sequence of events, i.e. receptor binding, engulfment (together with extracellular milieu), gradually decreasing pH in endocytic vesicles and other changes of the ionic environment is certainly important for a successful infection. To dissect these processes, we used different approaches for the evaluation of structural modifications occurring in the capsid and the genome, aiming to identify individual roles for each of them in preparation for RNA release.

## Results

### The ionic milieu strongly impacts on the stability of the RV capsid

**Fig. 1:**
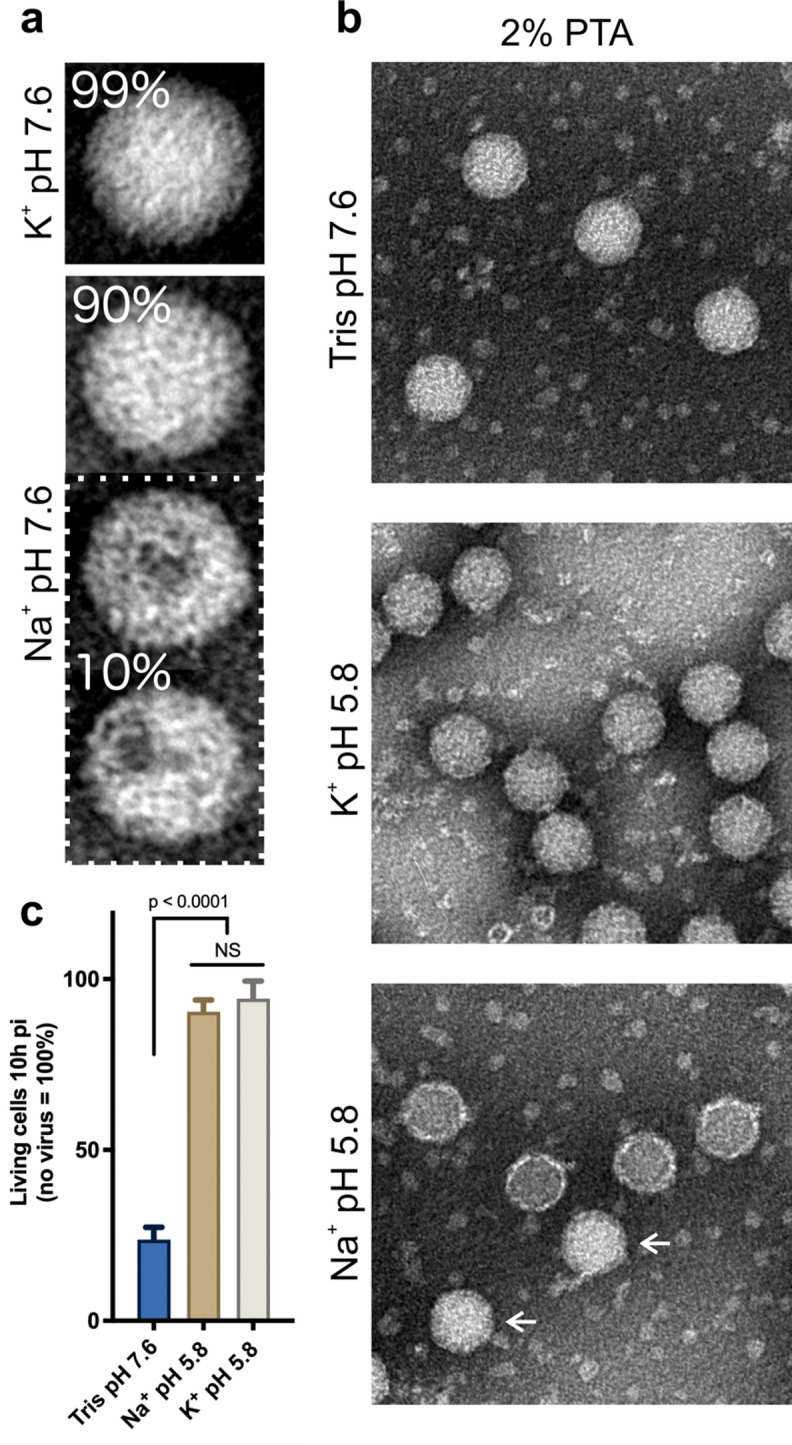
Effect of sodium phosphate buffer and potassium phosphate buffer on RV-A2. (**a**) Aliquots of purified RV-A2 were incubated overnight in Na^+^ or K^+^ phosphate buffer (pH 7.6) as indicated at 25°C and applied to carbon-coated electron microscopy grids, contrasted with 2% PTA, and imaged. The different particles were counted visually, and representative particles are displayed in (**a**). For the incubation in NaPB two examples of particles missing part of the capsid are shown (**a**, lower panel). No such particles were seen in KPB. (upper panel **a**, compare to **b**) Purified RV-A2 was incubated in 50 mM Tris-HCl pH 7.6 (control native virus) (upper panel) and KPB (middle panel) or NaPB (lower panel) (both at pH 5.8) for 1 h at 4°C, neutralised by addition of NaPB or KPB (pH 7.6) and stained with 2% PTA as indicated. In the lower panel non-converted, apparently native particles are indicated with arrows. (**c**) Aliquots of virus, similar as in **b**, were mixed with infection medium and added to semi-confluent HeLa cells in a 24-well plate (≈ 5 particles per cell) and incubated for 10 h. Cells were fixed with 1% formaldehyde and stained for 1 h with 0.2% (w/v) crystal-violet in 150 mM NaCl. Crystal-violet binds life cells with an intact membrane that remain adherent; cells lysed because of infection are lost during the subsequent extensive washes. Thus, the stain provides a direct read-out of the number of viable cells. Plates were imaged with a Zeiss Axio Observer Z1 inverted light microscope and the number of stained cells counted with Fiji software. Quantification from five independent wells was related to the number of live cells (i.e. 100%) in a non-infected well. ‘NS’—non-significant.

Sodium phosphate buffer (NaPB) and potassium phosphate buffer (KPB) have dissimilar effects on the accessibility of QGRS for pyridostatin within the genomic RNA inside the virion^9^. Therefore, we first asked whether these different cations affected the permeability of the protein shell or the compactness of the quasi-crystalline RNA core^22^. RV-A2 was incubated for 12 h at 25 °C in NaPB and KPB, respectively, stained with phosphotungstic acid (PTA) and viewed by electron microscopy. Identically looking particles were identified visually and counted. In KPB, about 99% of the particles appeared native. However, in NaPB only ~ 90% of the particles were apparently native with the remaining ~ 10% lacking parts, presumably pentamers, from the capsid (Fig. 1a). This clearly reflects the higher stability of the virus in the presence of K^+^. Partial capsid disassembly was described earlier for echovirus 18, a member of the genus *Enterovirus* B^6^ kept in phosphate buffer with high Na^+^ and low K^+^ concentration. The reduced capsid stability of RV-A2 in the presence of sodium ions was confirmed by using PaSTRy^19,23^ and nanoDSF^19^ (Supplementary Fig. 1). At elevated temperatures (Supplementary Fig. 1 green region), a resurgence of the SYTO 82 signal can be observed toward the temperature assumed to disintegrate pentamers^24^. The latter seems not to be affected by the type of cation.

The generation of RV subviral particles depends on the ionic composition of the incubation buffer, temperature and time of incubation. To test the impact of the metal cations present in the acidic buffer on the conversion from native virus to A particles, we incubated RV-A2 in NaPB or KPB, respectively, adjusted to pH 5.8, at 25 °C for 1 h and promptly neutralised. The samples were applied to glow-discharged carbon-coated EM grids, negatively contrasted with PTA, and viewed under the electron microscope. The acidic NaPB dismantled the majority of the native virus; remarkably, fragments were seen in close vicinity to each other, which strongly suggests that they originated from the same viral particle (Supplementary Fig. 2 red circle). On the other hand, the acidic KPB seems to not affect the overall stability of the viral particles. Aiming to slow the above disintegration, we repeated the incubations at 4 °C (Fig. 1b). RV-A2 in NaPB did not become disrupted but rather transformed into PTA-permeable A particles (lower panel) recognizable by the electron-dense interior, as compared with the native control virus (kept in Tris-HCl buffer pH 7.6; upper panel). In KPB the particles appeared indistinguishable from native virus (upper panel). Nevertheless, similarly to the A particle (as generated in NaPB), these particles lacked infectivity, when compared to RV-A2 incubated in neutral Tris-HCl buffer (Fig. 1c). The differences in the outcome of the negative staining with PTA suggests that incubation of native virus in acidic Na^+^ or K^+^ phosphate buffers gives rise to distinct structural differences in the resulting subviral particles.

### E0 particle characterization

Liu et al.^11^ identified a conformational intermediate occurring during capsid expansion, which they called ‘E1 particle’; this particle subsequently gave rise to the A particle that quickly released the RNA converting into an (empty) B particle. In contrast, the intermediate we identified here was very stable; it appeared to not suffer further modifications and, in vivo, might be a dead end in the infection process i.e., leading to abortive uncoating/infection. We called this intermediate ‘E0 particle’.

**Fig. 2:**
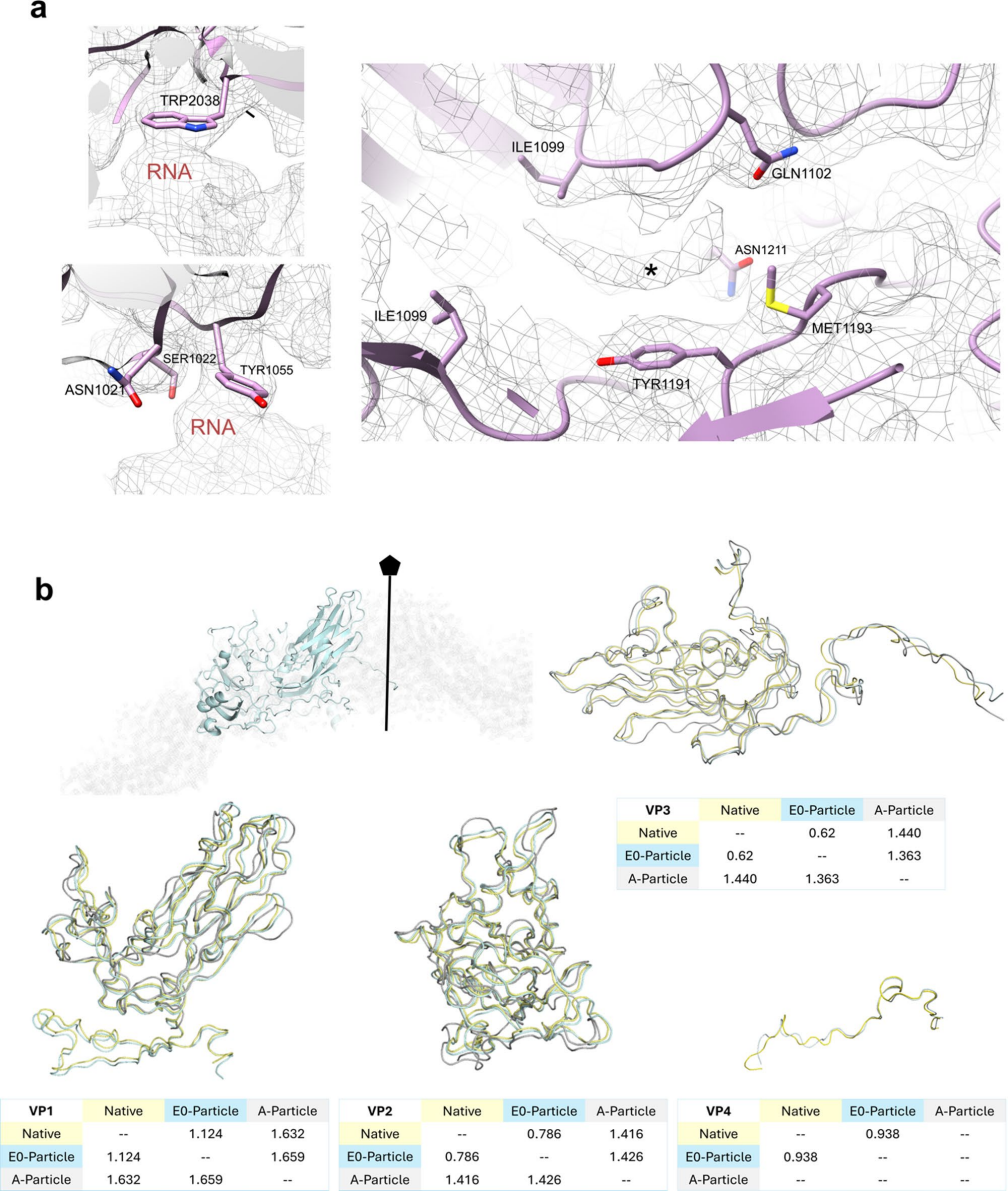
E0 particle features. (**a**) Interactions between capsid amino acid residues and RNA (left panels) and the volume inside the pocket (right panel—asterisk) presumably myristate (i.e. the pocket factor). (**b**) Superposition of capsid proteins (VP1-4) from E0 (blue), the native (yellow), and the A particle (grey) atomic models. The tables (under each model) present RMSD values (in Å) calculated by aligning each capsid protein individually using PyMOL (Version 3.0.0).

Solving the structure of the E0 particle formed in KPB (pH 5.8) to 3.2 Å (EMD 50930) allowed building an atomic model (PDB 9G0B). To address whether E0 was different from the A particle, we evaluated the interface between protein capsid and RNA, the occupancy of the hydrophobic pocket, and the presence of VP4 (Fig. 2a). These are structural hallmarks of the different viral particle conformations. The RV-A2 capsid possesses many contact points with the genome, with the best described amino acid residues TRP2038^19^ and ASN1021/SER1022/TYR1055^13^. We observed that only the aromatic rings interacted with the RNA in the E0 particle. The density inside the pocket was similar to that seen in native RV-A89^17^ and the RV-A2 crystal structure (PDB: 1FPN^25^, and was tentatively identified as lauric acid (C_12_ fatty acid), likely serving as the natural pocket factor. Furthermore, the presence of the pocket factor prevented the side chain of MET1193 from moving into the pocket, as is seen in RV-A89 devoid of the pocket factor^17^.

Inside the capsid, the genomic RNA is still fully retained and densely packed in the E0 particle. The 3.2 Å map did not allow tracing of the RNA genome. However, the capsid interior shows a bulk density consistent with tightly packed RNA, similar to the native virion. Importantly, no RNA strands are seen protruding or organizing into distinct helices in the E0 map, implying that the genome has not begun to exit. Structurally, the capsid of E0 is largely similar to the native virion.

To additionally compare E0 with native RV-A2 and the A particle, we used VIPERdb tools^26^ on the molecular models present in the data base (Table 1). This demonstrated that the capsid of the E0 particle is expanded, when compared, to the native virion but not as much as the A-particle, supporting the observed inaccessibility of the E0 core to PTA (Fig. 1b). Noteworthy is the reduction of the capsid thickness observed in the A particle calculated by subtraction of inner radius from outer radius. The S-score provides additional validation for the differences mentioned above^27^. Superposition of VP1-4 from three atomic models (native: PDB 3VDD^28^, A particle: PDB 4L3B^13^, and E0 particle: PDB 9G0B) revealed that the conformation of the E0 VP backbones is more similar to the backbones of the VPs in the native virion than to those in the A particle (Fig. 2b). Alignment with PyMOL demonstrated that VP1 and VP4 from E0 were more different from the native particle than VP2 and VP3. Overall, the E0 particle was more similar to the native than to the A particle (Fig. 2b; Table 1, and Supplementary Table 1).

**Table 1:** Comparison between different uncoating intermediates using VIPERdb v3.0^26^.

Particle	Radius inner (Å)	Radius outer (Å)	Difference (Å) = capsid thickness	S-score resp. to 9G0B
Native (3VDD)	100	159	59	0.889
E0 (9G0B)	103	165	62	1
A (4L3B)	114	167	53	0.663

### The RV-A2 RNA core alters its conformation in NaPB, but not in KPB

**Fig. 3:**
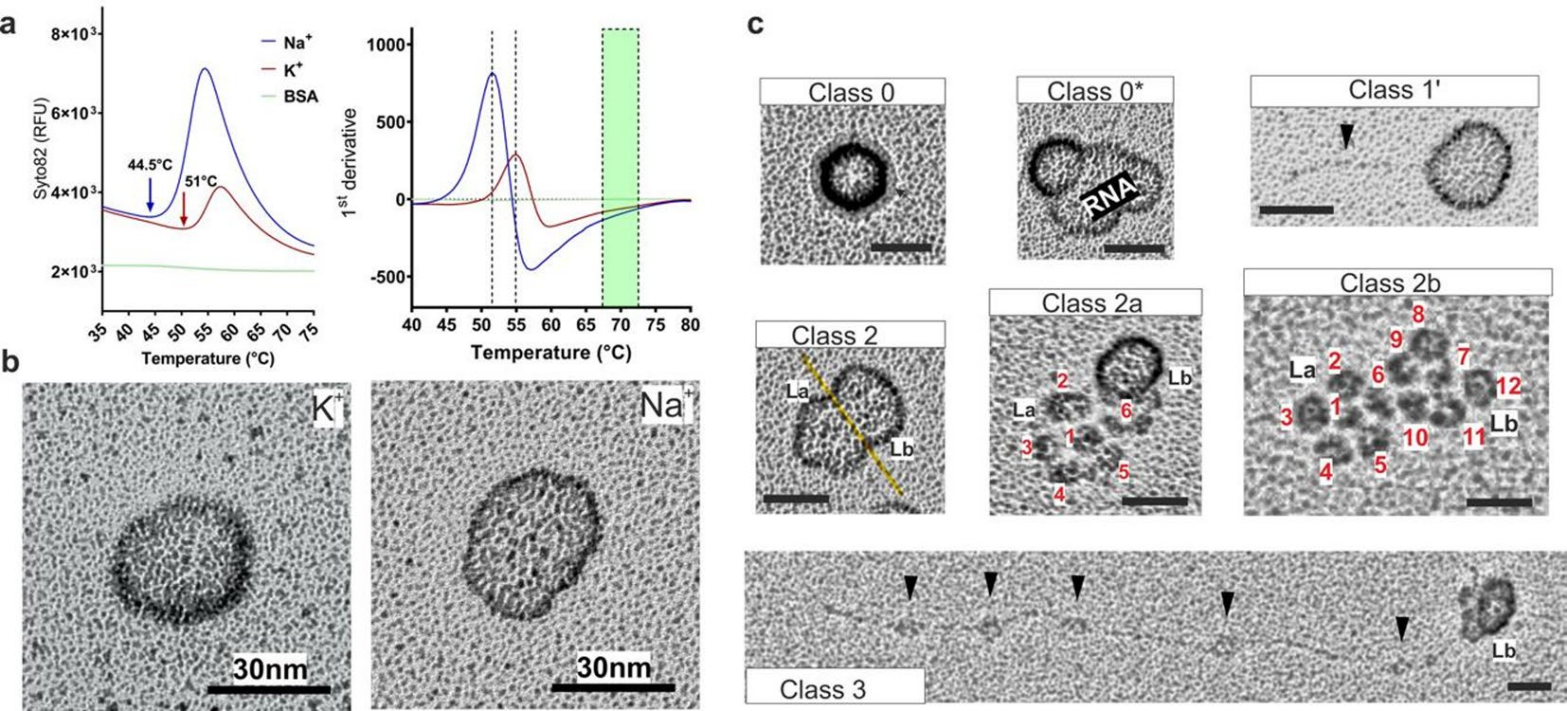
Analysis of RV-A2 RNA cores in NaPB or KPB. (**a**) PaSTRy assay revealing the accessibility of the RNA cores to the double-strand binding probe Syto-82. BSA was used as a non-binding control. The respective temperatures, where access of Syto-82 to dsRNA was starting are depicted with arrows and broken vertical lines, respectively. The green bar indicates complete denaturation of RNA double strands. (**b**) Rotary shadowing EM of RNA cores in KPB and NaPB. Note the more spherical appearance in KPB. (**c**) RNA cores in NaPB buffer were classified according to the degree of condensation/extension. Class 0 include incompletely digested protein capsids (arrow). Class 0* incompletely digested protein capsids with a largely open shell showing condensed, escaping RNA. Class 1’ includes mostly homogeneous cores as in (**b**) but with an escaping RNA strand, possibly the poly-A tail (arrowhead^7,8^). Class (2) are slightly extended RNA cores with two parts, La and Lb. The equatorial region between these divisions is marked by a yellow line. Class (2a) one of the two portions remains condensed (Lb) and the other one (La) appears to be disrupted into 6 ‘nodes’ or spheroids, presumably originating from interactions with pentamers. Class (2b) the two parts (La and Lb) are disrupted into 12 spheroids. Class (3) RNA core with a sector remaining condensed (Lb) and the other one partially or totally extended (short-ranged interaction nodes are indicated by arrowheads). All bars 30 nm. More examples are shown in Supplementary Fig. 4.

The above data suggest that pH and cationic composition of the milieu both impact on the uncoating process. To investigate the role of the extremely compact RNA genome separately, RV-A2 capsid proteins were removed via gentle proteolysis in NaPB or KPB, resulting in protein-free RNA cores^9^. We then accessed these cores for their accessibility to the double-strand binding fluorescent probe SYTO82; indeed, in KPB the fluorescence was clearly lower than in NaPB, which might be due to lower accessibility of the double stranded regions by preservation of RNA compactness of the cores (Fig. 3a). Bovine serum albumin was included as control to exclude that the signal stems from interaction of SYTO82 with protein fragments remaining from protein capsid proteolysis. Temperature ramping also showed that the peak of fluorescence was shifted from 44.5 °C, in NaPB, to 51 °C, in KPB. Such cores obtained in NaPB and in KPB were then subjected to rotary shadowing followed by EM. This revealed a clear difference (Fig. 3b and Supplementary Fig. 3).

The above findings suggest that the RNA cores in NaPB are more flexible and extended allowing diffusion of the dye into the cores. This hypothesis is supported by the identification of diverse forms of unravelling of the RNA that were visually classified according to their degree of condensation/extension (Fig. 3c and Supplementary Fig. 4; from Class 1’ to Class 3). Class 0 included incompletely digested viruses with Class 3 showing partially or totally extended RNA. The size of the thread-like structure in the Class 1’ is compatible with the size of the poly-A tail (60–100 nm). Class 2 suggests the existence of two segments that are formed by 6 parts or ‘nodes’ each, an entire virion would thus have 12 nodes in total. Notably, this is also the number of pentamers in the capsid. Detailed evaluation of the images corresponding to Class 3 revealed that the spheroids on the RNA strand might represent short-ranged RNA interactions (arrowheads), diverging from current models of genome organization within the capsid. Quantifying ~ 600 particles in eight randomly selected micrographs (similar to Supplementary Fig. 3, upper panel) yielded the following class distribution: class 0, 0–2%; class 1, 81–85%; class 2, 3–7%; and class 3, 9–13%.

## Discussion

Previous work indicated the importance of the cations most abundant in the biological environment in which the viral replication cycle of picornaviruses takes place. In 1986 Koch and Koch^29^ showed that the concentration of ions, in particular K^+^, inside the poliovirus capsid considerably exceeds the intracellular values. Later, Irurzun and Carrasco^14^ demonstrated, by using valinomycin, that high K^+^ concentrations impede poliovirus uncoating until it is replaced by Na^+^ in early endosomes. Aiming to better understand the subtlety of the uncoating process we investigated the effect of these ions on the protein capsid and the RNA conformation, permeability, and infectivity of the RV-A2 genome during uncoating in vitro. Incubation of RV-A2 in KPB at pH 5.8 revealed a novel uncoating intermediate (the E0 particle) with a slightly expanded shell and less permeability to PTA than the A particle. PTA has a relative molecular mass in its dehydrated form of 2880.05 g mol^−1^, whose diffusion into the highly compacted RNA might be limited.

To situate the E0 particle within a physiological context: (i) Rhinovirus genome release occurs from endosomes possessing a Na^+^/K^+^ exchanger, which replaces luminal Na^+^ with cytosolic K^+^ during acidification. (ii) If K^+^ influx precedes sufficient acidification, the endosomal lumen becomes K^+^-rich before the pH can trigger RNA uncoating. (iii) Consequently, virions are maintained in the E0 state and RNA release is inhibited. Note that K^+^-loading arrests poliovirus uncoating in cells exposed to valinomycin or cultured in high-K^+^ medium^14^. Therefore, E0 particles likely mark an abortive branch of the entry pathway that might function as a host-defence. This apparent ‘dead end’ in infection may partially account for the high particle-to-PFU ratio (about 1000:1) observed in RV^30^. Only virions within the correct pH/[Na^+^/K^+^] concentration window transition beyond E0 to productive uncoating.

Additionally, the E0 particle has a distinct capsid-RNA interface when compared to the A particle. Of note, whereas in the A particle several contacts between RNA and the inner wall of the capsid are seen, in the E0 particle only the stacking interactions of the RNA with aromatic residues are clearly discernible. The resistance of a highly compacted genome against untying and expansion was recently elegantly demonstrated in a bacteriophage^31^; using optical tweezers to measure the unravelling speed of the phage genome revealed that addition of Na^+^ resulted in faster extension of the genome. Thus, stabilization of the nucleic acid core by K^+^ and its destabilization/loosening by Na^+^ and the associated hydration might be a general phenomenon in genome release by viruses.

As exemplified by RV-B14, the rhinovirus RNA core is as compact (i.e. its value of compactness, V_m_ = (V_container_ (Å^3^)/M_r(contents)_(Da)) as crystalline (U((UA)_6_)A)_2_ duplex RNA^32^. The expansion of the E0 particle with respect to the native virion during uncoating might originate from some minor rearrangements of the RNA brought about by changes in the ionic environment and solvation. A small RNA rearrangement was seen for coxsackievirus B3 at points of interaction with receptor-decorated nanodiscs. Although it is not clear whether the RNA or the protein capsid are the main players in the expansion, this work suggests that a small gain in volume gives the RNA substantially more freedom to move^16^. Potassium ions are the main enterovirus RNA backbone neutralisers^29^ and Na^+^-driven hydration enhances dynamics and mobility of the RNA^33^. We suggest that during endocytosis (and as shown here, in vitro uncoating), the potassium ions become replaced by sodium ions and water; for a review on the ionic composition of endocytic vesicles see Ref.^18^. In our hypothesis, the crystalline RNA remains stable in potassium ions but gains plasticity in sodium ions. This is supported by the higher solvation energy (charge/size) ratio of Na^+^; reviewed by Draper^34^. The change in the plasticity of the RNA core is supported by the recently reported effect of pyridostatin on RV RNA cores^9^. Just as a curiosity, when Na^+^ in glass is being replaced with K^+^ it becomes considerably more resistant against breakage^35^.

Notwithstanding these in vitro findings, in the cellular environment, complete RNA ejection seems to also depend on cellular proteins, including myosin Va and Vb^36^. The 12 spheroids observed upon incubation in NaPB (Fig. 3c) go hand in hand with the finding that the poliovirus genome immobilized on beads sequestered pentamers but not empty viruses^37^. Similar findings were made with parechovirus^38^.

Tracking of poliovirus RNA labelled with SYTO82 revealed a rapid decline in the fluorescent signal following endocytosis indicating the ejection of RNA as a single strand^39^. On the other hand, ‘long umbilical connectors’ were implicated in the passage of the RNA from the virus through the endosomal membrane^40^ suggesting that the RNA remains or becomes covered with protein during the process. RNA exit from the virion might occur either as a single strand (see above and Ref.^8^) possibly through a hole at a two-fold axis of the virion opening upon conversion into the A particle^41^ or as bulk upon loss of a portion of the capsid, e.g. one or more pentamers^6^. The latter is compatible with the particles shown at the bottom of our Fig. 1a. Both distinct ways of RNA ejection are not necessarily mutually exclusive. From our observations, as the RNA expands due to the replacement of potassium ions with sodium ions and water, we propose that the lower stability of the surrounding capsid directs the RNA to escape through a pore at the two-fold axis (in a more stable capsid, e.g. poliovirus) or to push out parts of the capsid (in a less stable capsid, e.g. echovirus).

In conclusion, we suggest that the K^+^-ions neutralising the negative charges of the phosphate backbone of the RNA within the virion become exchanged for Na^+^ and water within a slightly acidic milieu in early endosomes, which might lead to a small expansion of the RNA core and a modification of the RNA/protein interaction, as demonstrated in our present report. This would initiate the final acid-driven conversion of the E0 particle to the A particle and finally to the B particle. Our rotary shadowing EM images of various stages of the RNA release process and, in particular of the unravelling of the compact and spherical RNA core, suggest that the RNA forms ‘nodes’ or spheroids of short-range interacting strands interspaced with segments of low-complexity secondary structure. Evidence for degenerate repetitive sequences interacting with the capsid proteins has been presented for a parechovirus^38^ and recently for a rhinovirus^2,3^. At a first stage of uncoating, the RNA would still be connected to pentamers. These would remain close to each other because of their being linked via poorly structured RNA. In the absence of protein, this RNA would then expand and manifest as nodes or pearls on a string as clearly seen in our rotary shadowing EM images. We believe that our work adds a new dimension to the complicated and still ill-understood process of RNA release into the cytosol of the host cell.

## Materials and methods

### Cells and virus

HeLa Ohio cells were originally obtained from ATCC and maintained in DMEM (Merck), supplemented with 10% FBS (Life Technologies, Carlsbad, CA, USA), and 1% penicillin (Merck, New York, NY, USA) and streptomycin (Merck). Cells were kept in a humidified, 5% CO_2_-containing atmosphere at 37 °C. For infection, the serum concentration was reduced to 2% FBS, and cells were incubated at 34 °C, the optimal growth temperature for RVs. RV-A2 was initially acquired from ATCC, propagated, and purified following the protocol detailed in^42^.

Purified RV-A2 (10 mg/ml) diluted in 100 mM sodium or potassium phosphate buffer (pH 5.8) were incubated for 1 h at 4 °C and neutralised by dilution (1:10) with 100 mM sodium or potassium phosphate buffer (pH 7.6). Aliquots were separated and subjected to negative staining as described in^43^ and remaining aliquots were mixed with infection medium (DMEM + 2% FBS) and added to semi-confluent HeLa cells in 24-well plates (MOI ≈ 5) and incubated for 10 h. RV-A2 kept in Tris-HCl buffer at 4 °C was used as infection control. Cells were fixed and stained with crystal violet solution and living cells were imaged using an inverted light microscope Zeiss Axio Observer Z1 and counted with Fiji software. Quantification was from five independent wells normalized by the number of alive cells in a non-infected well (100%).

### RV-A2 RNA morphology revealed by rotary shadowing electron microscopy

Purified RV-A2 in 100 mM potassium phosphate buffer (pH 7.6) or 100 mM sodium phosphate buffer (pH 7.6) was incubated with proteinase K for 12 h at 4 °C, protocol detailed in^9^. The samples were subsequently diluted 1:1 in spraying buffer (200 mM ammonium acetate and 60% (v/v) glycerol, pH adjusted to 7.6). Samples were immediately sprayed onto freshly cleaved mica chips (Agar Scientific, Birchanger, Essex, UK) and quickly transferred into a BAL-TEC MED020 high vacuum evaporator equipped with electron guns. While rotating, samples were coated with 0.6 nm platinum (BALTIC) at an angle of 7°, followed by 6 nm carbon (Balzers, Liechtenstein) at 90°. The replicas were floated off the mica chips, picked up on 400-mesh Cu/Pd grids (Agar Scientific), and inspected in an FEI Morgagni 268D TEM (Thermo Fisher Scientific) operated at 80 kV. Images were acquired using an 11-megapixel Morada CCD camera (Olympus SIS, Münster, Germany).

### PaSTRy of RV-A2 RNA

Purified RV-A2 in 100 mM potassium phosphate buffer (pH 7.6) or sodium phosphate buffer (pH 7.6) was incubated with proteinase K for 12 h at 4 °C. Samples were kept at 4 °C and mixed with SYTO 82 to a final concentration of 5 µM, and the volumes were adjusted to 70 µL with respective phosphate buffers. Three 20 µL aliquots from each of these samples were dispensed into the wells of a thin-walled PCR plate, the temperature was ramped from 25 to 95 °C at 1.5 °C/min, and SYTO 82 light-up fluorescence was recorded. Six independent measurements were made for each condition. Data were rendered as a dot plot revealing the temperature at which the RNA becomes accessible for SYTO 82 binding.

### Cryo-EM sample preparation and image collection

The RV-A2 sample was prepared as above. Four microliters of purified RV-A2 at 1 mg/ml in 5.84 mM K_2_HPO_4_, 94.16 mM KH_2_PO_4_ (pH 5.8) was applied onto glow discharged (30 s, 25 mA) copper Quantifoil grids (R2/2 200 mesh). After application, followed by incubation for 10 s, the sample was plunge-frozen in a liquid ethane at liquid nitrogen temperature using a Leica EM GP (Leica) set to 80% humidity and 293 K. Vitrified samples were imaged on a Glacios TEM (ThermoFisher Scientific) operating at 200 kV, equipped with a field emission gun (X-FEG) and FEI Falcon III. In total 246 movies were recorded in integrating mode at 100,000x nominal magnification (physical pixel size 0.998 Å, consisting of 60 frames over 1 s (total electron exposure of 60.0 e/Å^2^, corresponding to 1.0 e/Å^2^ per frame), and images were recorded with using ThermoFisher Scientific EPU data collection software, with a defocus range between 0.5–2.5 μm.

### Cryo-EM image processing and atomic model building

Single-particle analyses were performed using Relion (v4.0). The total of 246 movies yielded ~ 16,372 particle projections, which were initially auto picked and extracted (450 × 450-pixel extraction box). These particles underwent exhaustive rounds of 2D/3D classification in RELION, and ultimately ~ 9825 particles were retained for the final 3D reconstruction. During refinement, icosahedral symmetry (I2) was imposed to exploit the virus’s 60-fold symmetric capsid. Image processing was carried out using RELION 4.0 for particle selection, 2D/3D classification, and gold-standard refinement, with CTF parameters determined by CTFFIND 4.1. (Movie alignment and particle polishing were part of the RELION workflow, ensuring optimal signal recovery). The final 3D map was reconstructed by RELION’s auto-refine with icosahedral point-group symmetry, converging on a high-resolution density map for the E0 particle.

The final E0 cryo-EM map achieved a resolution of 3.2 Å, based on the gold-standard FSC 0.143 criterion. (The FSC = 0.5 threshold corresponded to ~ 3.46 Å, and the 0.5-bit ‘half-bit’ criterion to ~ 3.15 Å, indicating a robust high-resolution reconstruction.) The map was deposited with a recommended contour level of 0.05 (fraction of maximum) and was sharpened to accentuate high-frequency features. The quality of the map and fit of the atomic model were confirmed by multiple validation metrics. Independent half-map refinement (two half-data sets) was used to prevent overfitting, and the FSC curve from the two half-maps showed the typical gold-standard behaviour.

Model building was started by fitting the PDB 3VDD model into electron microscopy maps using the fit-in-map tool in UCSF ChimeraX (v1.8). Several rounds of real-space refinement using Phenix (v1.21) and REFMAC (Coot v0.9.5) were performed. Further interactive refinement was performed with Coot (v1.1.08). The MolProbity (Phenix v1.21) was used to validate model geometries. The resulting model-to-map agreement is very good: about 90% of all atoms in the final model are encompassed by density at the chosen contour level (0.05), and the average map Q-score per atom is ~ 0.60. Overall, the validation report confirms robust model geometry and fit, with no signs of overfitting: real-space correlation of the model to the map is high, and cross-validation using one half-map for refinement and the other for model validation supports the reliability of the structure. UCSF Chimera (v1.8) was used for visualizations and analysis.

Examples of map quality are demonstrated in Supplementary Fig. 5.

## Supplementary Information

Below is the link to the electronic supplementary material.

Supplementary Material 1

## Data Availability

Cryo-EM density maps resolved in this study have been deposited in the Electron Microscopy Data Bank (EMDB) (http://www.emdataresource.org/EMD-50930) under accession code: EMD-50930. The corresponding coordinates have been deposited in the Research Collaboratory for Structural Bioinformatics Protein Data Bank (RCSB PDB) (http://www.rcsb.org/structure/9G0B) under accession code: 9G0B.
