# Supplementary material for: New rhinovirus uncoating intermediate reveals how sodium versus potassium ions influence RNA release
Source: Sci Rep. 2025 Oct 21;15:36768. doi: 10.1038/s41598-025-20627-0 (PMC12541096; doi:10.1038/s41598-025-20627-0)
Supplement: Supplementary file 1 — Supplementary Material 1 [file 41598_2025_20627_MOESM1_ESM.pdf]

New rhinovirus uncoating intermediate reveals how sodium versus potassium ions influence RNA release

Antonio Real-Hohn* and Dieter Blaas*

Centre for Medical Biochemistry, Max Perutz Labs, Vienna BioCenter, Medical University of Vienna, Vienna, Austria

*Corresponding authors

E-Mail address: antonio@realhohnlabs.com, dieter.blaas@meduniwien.ac.at

Supplementary Material List:

Supplementary Table 1

Supplementary Fig. 1

Supplementary Fig. 2

Supplementary Fig. 3

Supplementary Fig. 4

Supplementary Fig. 5

Supplementary Table 1. Comparison between N, E0, A, and B particles.

Feature	Native (N)	Expanded (E0)	A particle (A)	B particle (B)
Capsid expansion (Δouter radius vs N)	–	+ 6 Å (165 Å vs 159 Å)	+ 8 Å (167 Å)	~+ 8 Å (similar to A; from the literature)
VP4 present?	**Yes**	**Yes**	**No** (lost during expansion)	**No**
Pocket-factor present?	**Yes**	**Yes**	**No** (pocket is empty/collapsed)	**No**
Genome present?	**Yes**	**Yes**	**Yes** (but poised for release)	**No**
PTA penetrates shell?	No (capsid impermeable)	No / very little	**Yes** (interior stains dark)	**Yes**
Infectious?	**Yes**	No	No (entry intermediate)	No
Typical role	Stable extracellular virion	Abortive pathway intermediate	Proven uncoating intermediate	End-point, empty shell

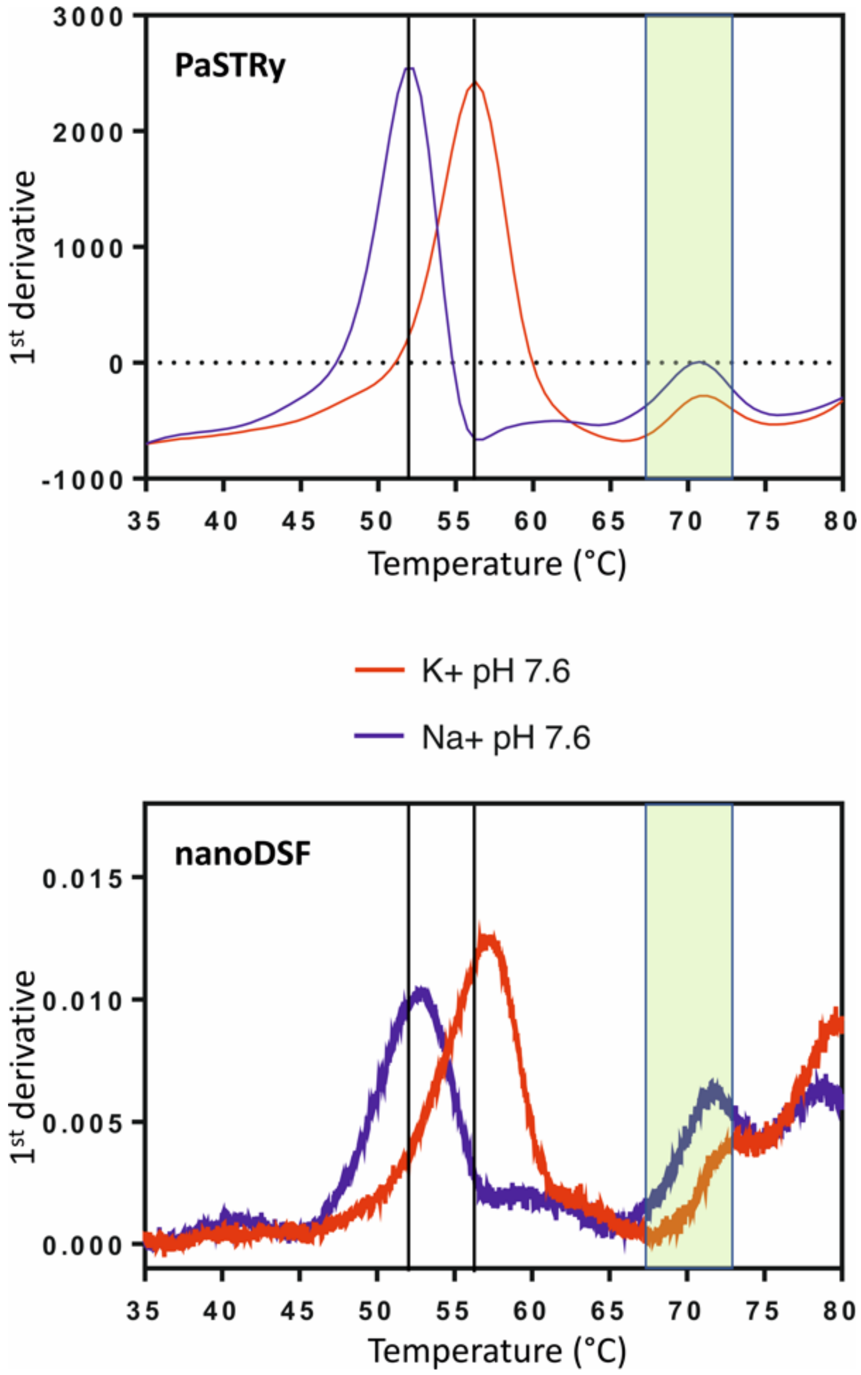

Supplementary Fig. 1 PaSTRy and nanoDSF RV-A2 in NaPB vs KPB. PaSTRy – Purified RV-A2 (1 mg/ml) in 100 mM KPB (pH 7.6) or 100 mM NaPB (pH 7.6) were mixed with SYTO 82 (final concentration 5 mM). Three aliquots of each experimental condition were disposed into the wells of a thin-walled PCR plate, the temperature was ramped from 25–95 °C at 1.5 °C/min. Samples were excited at 541 nm, and emission intensity was determined at 560 nm at each 0.5 °C temperature increase. The recorded SYTO 82 fluorescence signal and the first derivative of it were plotted with GraphPad Prism 6.01 against the temperature. nanoDSF – Purified RV-A2 (1 mg/ml) in 100 mM KPB (pH 7.6) or 100 mM NaPB (pH 7.6) was analysed by nanoDSF heating from 25 to 95 °C with a ramp rate of 1 °C/min. Samples were excited at 280 nm, and the intensity of the TRP emission at 330 and 350 nm was recorded for every ~0.02 °C temperature increase. The first derivative from these curves was generated with GraphPad Prism 6.01 and plotted against the temperature. The green area on the plots represents the region of full dismantling of the capsid. A similar dismantling at high-temperature was described for poliovirus in [1].

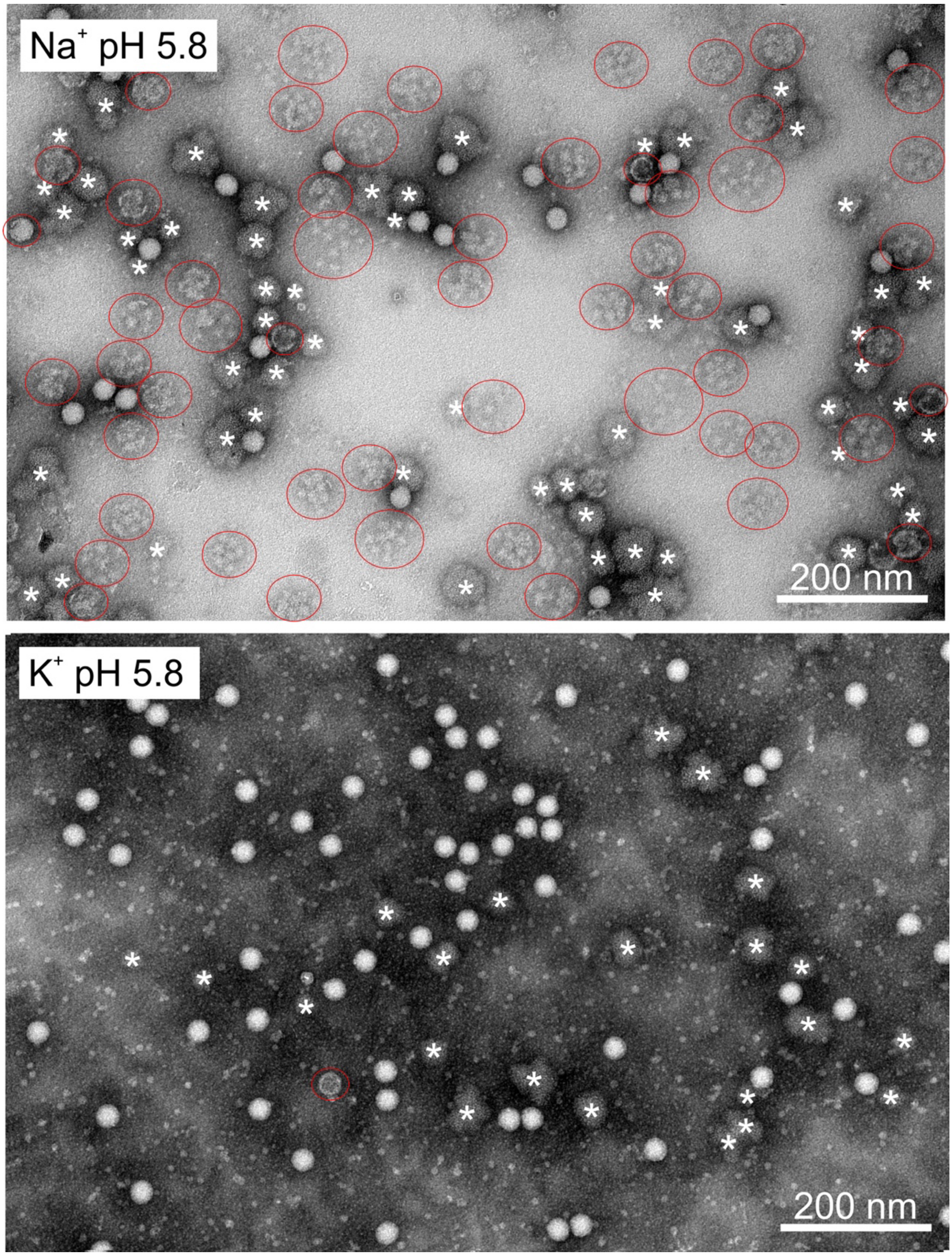

Supplementary Fig. 2, RV-A2 incubated in Na^+ or K^+ phosphate buffer (pH 5.8) at 25 °C for 1 hour. Purified RV-A2 (10 mg / ml) diluted to 1 mg / ml in 100 mM NaPB or KPB (pH 5.8) were incubated for 1 hour at 25 °C and neutralised by addition of 1/100 volume of 100 mM NaPB or KPB (pH 7.6). Disrupted capsids are marked with red circles. Contaminating material is marked with a white asterisk.

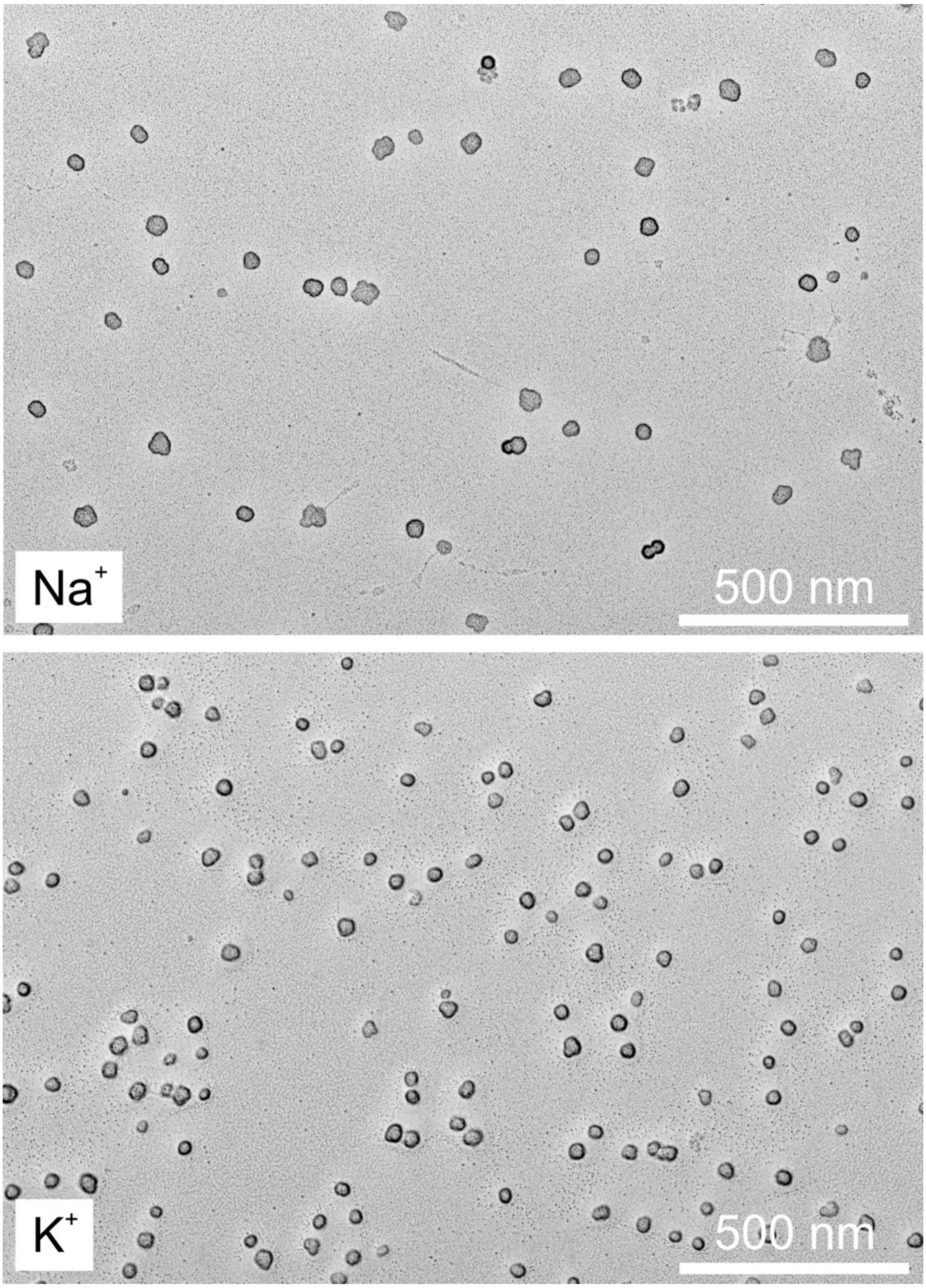

Supplementary Fig. 3 RV-A2 RNA core in Na^+ or K^+ phosphate buffer. Purified RV-A2 (1 mg / ml) in 100 mM NaPB or KPB (pH 7.6) was incubated with proteinase K for 12 h at 4 °C and divided into two aliquots. The samples were mixed 1:1 with 200 mM ammonium acetate, 60% (v/v) glycerol, sprayed onto freshly cleaved mica chips, and coated with 0.6 nm platinum at an angle of 7° under high vacuum. Replicas were floated onto carbon-coated grids and imaged under an FEI Morgagni transmission electron microscope. Note more heterogeneity in NaPB with some of the particles showing segments of presumably ssRNA.

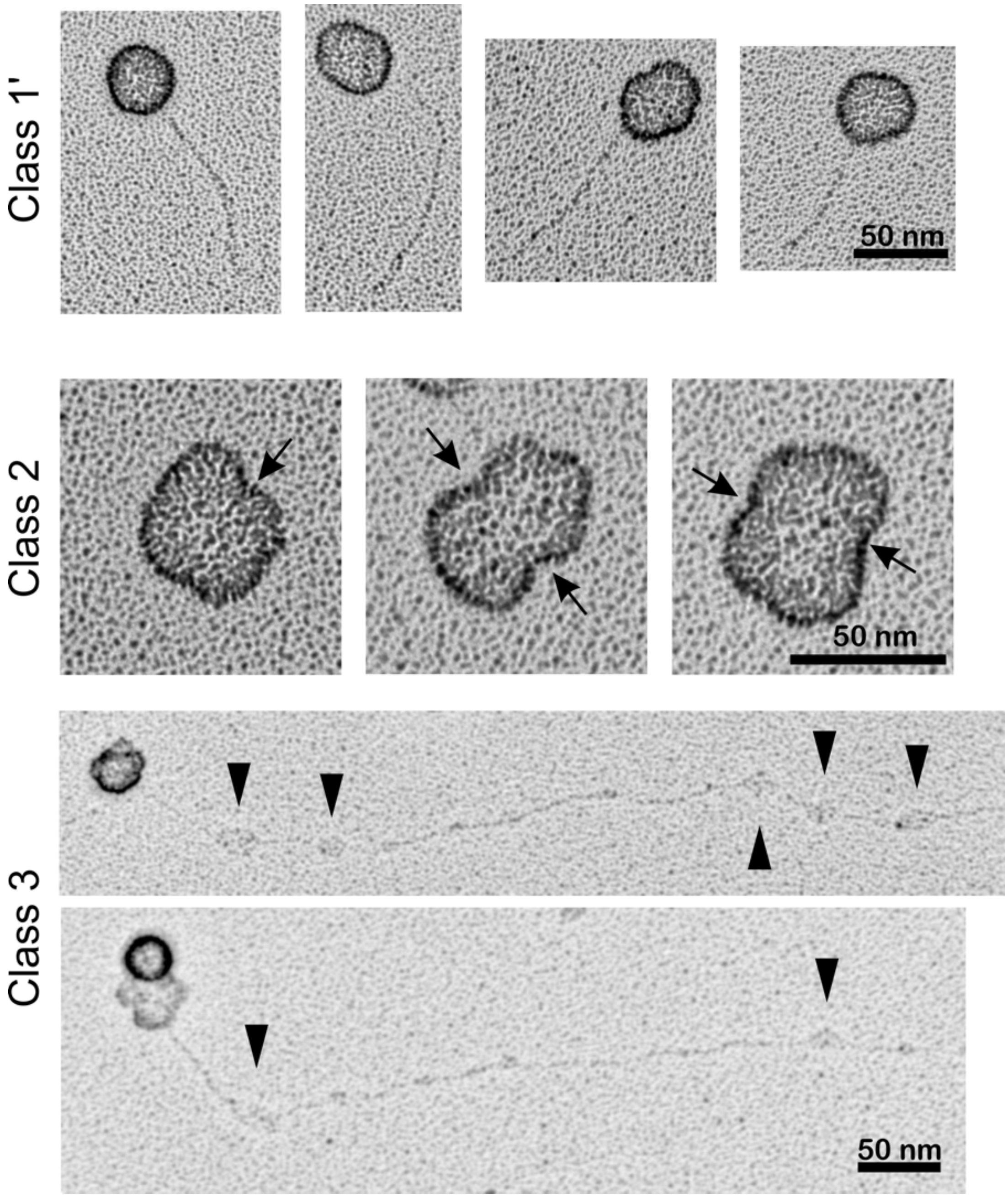

Supplementary Fig. 4 Classes of RV-A2 RNA unfolding states. Purified RV-A2 (1 mg / ml) in 100 mM NaPB (pH 7.6) was incubated with proteinase K for 12 h at 4 °C. The sample was mixed 1:1 with 200 mM ammonium acetate, 60% (v/v) glycerol, sprayed onto freshly cleaved mica chips, and coated with 0.6 nm platinum at an angle of 7° under high vacuum. Replicas were floated onto carbon-coated grids and imaged under an FEI Morgagni transmission electron microscope. Class 1') encompasses condensed RNA with a segment of presumably ssRNA that might be the poly-A tail. Class 2) encompasses condensed RNA with two parts separated by an incision (equatorial region between these parts are marked with arrows). Class 3) encompasses partially condensed RNA as in Class2 but with one of the two parts being extended. The later appear to represent short-ranged interactions (arrowheads).

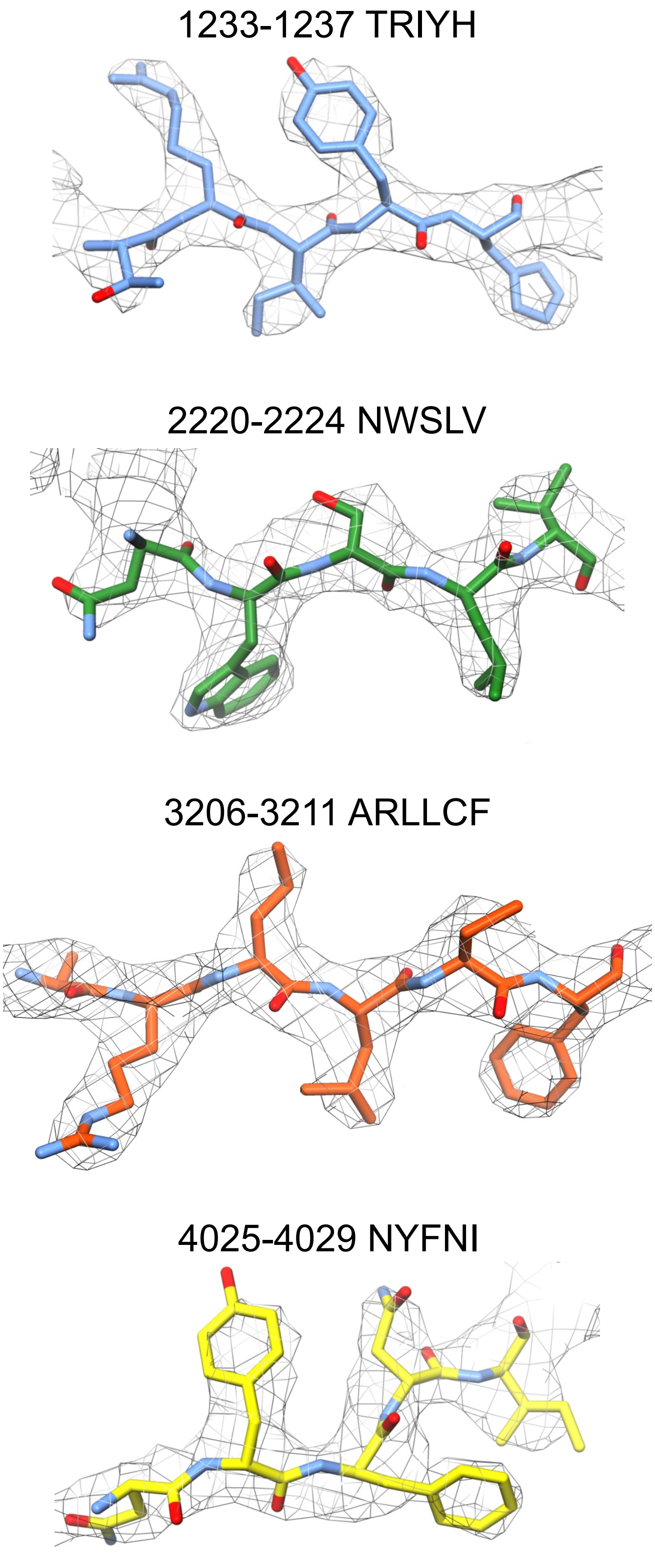

Supplementary Fig. 5 Cryo-EM Map Quality. Superimposition of coordinates (PDB 9G0B) and cryo-EM map (EMD-50930). Depicted residues from each VP and positioning are presented. VP1 – Blue, VP2 – Green, VP3 – Orange, VP4 – Yellow.
